# Desmosomes: emerging pathways and non-canonical functions in cardiac arrhythmias and disease

**DOI:** 10.1007/s12551-021-00829-2

**Published:** 2021-09-04

**Authors:** Jing Zhang, Yan Liang, William H. Bradford, Farah Sheikh

**Affiliations:** Department of Medicine, University of California San Diego, 9500 Gilman Drive, La Jolla, CA 92093 USA

**Keywords:** Heart, Desmosome, Cardiac muscle, Cell-cell junction, Arrhythmias, Inflammation, Cardiomyopathy

## Abstract

Desmosomes are critical adhesion structures in cardiomyocytes, with mutation/loss linked to the heritable cardiac disease, arrhythmogenic right ventricular cardiomyopathy (ARVC). Early studies revealed the ability of desmosomal protein loss to trigger ARVC disease features including structural remodeling, arrhythmias, and inflammation; however, the precise mechanisms contributing to diverse disease presentations are not fully understood. Recent mechanistic studies demonstrated the protein degradation component CSN6 is a resident cardiac desmosomal protein which selectively restricts cardiomyocyte desmosomal degradation and disease. This suggests defects in protein degradation can trigger the structural remodeling underlying ARVC. Additionally, a subset of ARVC-related mutations show enhanced vulnerability to calpain-mediated degradation, further supporting the relevance of these mechanisms in disease. Desmosomal gene mutations/loss has been shown to impact arrhythmogenic pathways in the absence of structural disease within ARVC patients and model systems. Studies have shown the involvement of connexins, calcium handling machinery, and sodium channels as early drivers of arrhythmias, suggesting these may be distinct pathways regulating electrical function from the desmosome. Emerging evidence has suggested inflammation may be an early mechanism in disease pathogenesis, as clinical reports have shown an overlap between myocarditis and ARVC. Recent studies focus on the association between desmosomal mutations/loss and inflammatory processes including autoantibodies and signaling pathways as a way to understand the involvement of inflammation in ARVC pathogenesis. A specific focus will be to dissect ongoing fields of investigation to highlight diverse pathogenic pathways associated with desmosomal mutations/loss.

## Introduction

The desmosome is integral for maintaining structural integrity in tissues undergoing constant mechanical stress such as the heart (Najor 2018). The cardiac desmosome is composed of trans-membrane cadherins desmoglein-2 (DSG2) and desmocollin-2 (DSC2), which are anchored to the armadillo proteins plakoglobin (JUP) and plakophilin-2 (PKP2), and desmoplakin (DSP), which tethers the intermediate filament network desmin (Des) to the cardiac junction (Sheikh et al. 2009; Najor 2018). Mutations in desmosomal genes are linked to 50% of cases for the heritable cardiac disease, arrhythmogenic right ventricular cardiomyopathy (ARVC), revealing the importance of this structure in cardiac function (Delmar and Mckenna 2010; Vimalanathan et al. 2018). ARVC patients have a complex disease presentation, with some demonstrating electrical abnormalities in the absence of overt structural change, and others susceptible to severe tissue remodeling and inflammatory response (Vimalanathan et al. 2018; Gao et al. 2020). While the desmosome is conventionally linked to cellular adhesion and structural support, extensive work has focused on identifying new pathways that may contribute to the complex disease presentation. This review will highlight emerging areas of research suggesting desmosomal mutations/loss are linked to protein degradation mechanisms, arrhythmia-based channel function, and inflammation.

## Protein degradation mechanisms targeted at proteins of the cardiac desmosome

**Fig. 1:**
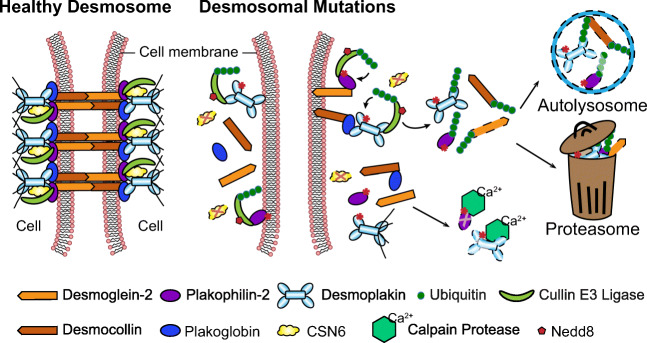
Schemata of protein degradation pathways at the cardiac desmosome. (a) In healthy cardiomyocytes, CSN6 interacts with the desmosomal complex (via DSP and PKP2) at the cardiac cell-cell junction. CSN6 inhibits activity of cullin E3 ligases by removing Nedd8 modifications to prevent desmosomal degradation/dissolution. The intact desmosomes tightly attach two adjacent cells together to maintain the mechanical integrity and proper electrical conduction. Calpain has limited accessibility to the cardiac desmosome. (b) Desmosomal mutations result in the destabilization of CSN6-desmosmal interaction. Absence of CSN6 allows attachment of Nedd8 and activates Cullin E3 ligases, which polyubiquitinate desmosomal proteins. Polyubiquitinated desmosomal proteins are cleared by proteasome or autolysosome, leading to desmosomal structural dissolution. Desmosomal mutations also increase the surface exposure to calpain and trigger their vulnerability to calpain-mediated degradation

Emerging new evidence has revealed that defects in protein degradation trigger the structural remodeling underlying the desmosomal disease, ARVC (Kirchner et al. 2012; Ng et al. 2019; Hoover et al. 2021; Liang et al. 2021). Protein degradation pathways are important toward maintaining cardiac function as they tag misfolded, mutated, damaged, and unneeded proteins for disposal (Lyon et al. 2013;Wang and Wang 2015). Given that desmosomal protein dissolution/loss is a central molecular alteration in desmosomal diseases, such as ARVC, several studies have shed light into the mechanistic underpinnings of how this loss may be triggered by connections to calcium-dependent, non-lysosomal proteolytic enzymes found in the cytosol as well as more directly via resident ubiquitin-proteasomal proteolytic machinery (Kirchner et al. 2012; Ng et al. 2019; Hoover et al. 2021; Liang et al. 2021) (Fig. 1).

### Calpain-mediated targeting of desmosomal protein degradation

Calpains are intracellular calcium-dependent cysteine proteinases that have been shown to be triggered by calcium overload and play a critical role in cardiac hemodynamic stress (Taneike et al. 2011); however, only recent studies have provided evidence for their role at the cardiac desmosome. Recent evidence highlight that the desmosomal proteins, PKP2 and DSP, contain putative calpain binding sites that when exposed through a subset of ARVC-related mutations, may trigger their vulnerability to calpain-mediated degradation (Kirchner et al. 2012; Ng et al. 2019; Hoover et al. 2021). In silico analyses alongside cell and bacterial expression studies of desmosomal mutant proteins provided key insights into demonstrating the vulnerability of a subset of ARVC mutations in PKP2 (C-terminus: C796R, S615F, C693fsX741, and K654Q) and DSP (N terminus: R415G, S299R, S442F, and S507F) to rapid protein degradation (and loss of cell-cell junction localization) due to increased surface exposure to calpain (Kirchner et al. 2012; Ng et al. 2019). In terms of human PKP2 mutations, further studies revealed that PKP2 mutant proteins were the target of calpain protease degradation, as calpain inhibition (ALLN, ZLL), could partially restore PKP2 protein levels (Kirchner et al. 2012). Proteasome, autophagy, and Golgi-endosome inhibitors were shown to have little effect on PKP2 protein levels in this context (Kirchner et al. 2012). Interestingly, calpain inhibition via MDL-28170 was shown to exhibit a very mild rescue effect of DSP protein levels with respect to the DSP R415G mutation (Ng et al. 2019). To more precisely validate the role of calpain in driving DSP-mediated protein degradation, molecular dynamic simulations revealed that the DSP L518Y mutation could serve as a means to block DSP calpain binding site accessibility, without causing secondary structural alterations (Hoover et al. 2021). Through this “molecular band aid” approach, it was shown in cell-based approaches that the DSP L518Y could successfully increase DSP protein levels in the context of DSP S422F and S507F mutations (Hoover et al. 2021). However, this approach was not sufficient to rescue DSP degradation in the context of the DSP S299R and R451G mutations as it was cited that this molecular alteration (L518Y) may not provide sufficient range to interfere with calpain accessibility (Hoover et al. 2021). Alternatively, there remains the possibility that additional mechanisms may be at play in driving DSP/desmosomal-mediated protein degradation.

### COP9-mediated targeting of desmosomal protein degradation

Constitutive photomorphogenesis 9 signalosome (CSN) is a multiprotein enzymatic complex consisting of eight subunits (CSN1-8), with known functional roles in controlling ubiquitin-mediated protein degradation as it has been shown to de-neddylate and inactivate cullin RING E3 ubiquitin ligases (Cavadini et al. 2016). Recent studies have implicated complex interactions between CSN subunits and desmosomal proteins (Najor et al. 2017;Liang et al. 2021), with a recent study implicating CSN6 as a resident cardiac desmosomal protein that selectively restricted cardiomyocyte desmosomal protein degradation and disease (Liang et al. 2021). A desmosomal-signalosome resident complex was identified in cardiomyocytes that encompassed structural interactions between DSP and PKP2 and the CSN enzymatic core subunits, CSN6 and CSN5, as well as CURLs (cullins 1 and 3) (Liang et al. 2021). Based on in silico studies, it was postulated that the spectrin domain in DSP and MPN domain in CSN6 may be the structural-signaling basis for this protein-protein interaction and key for desmosomal substrate recognition by CSN machinery (Liang et al. 2021). Using cardiomyocyte CSN6 knockout mice, it was shown that this structural-signaling axis was disrupted as CSN6-deficient hearts exhibited rapid and selective desmosomal protein degradation, which was triggered by loss of CURL-mediated control (hyper-neddylated cullins) as well as hyperaccumulation of ubiquitin and selective autophagy protein degradation machinery at the cardiac desmosome, ultimately resulting in desmosomal structural dissolution (Liang et al. 2021). Most poignant were studies performed in CSN6-deficient neonatal mouse cardiomyocytes that showed that the neddylation inhibitor (MLN4924) could alleviate the desmosomal protein loss in CSN6-deficient cardiomyocytes, and showcasing that desmosomal proteins are under neddylation control (Liang et al. 2021). The relevance of the cardiomyocyte CSN6-desmosomal complex in human cardiac disease was further shown as (i) cardiomyocyte-specific CSN6 knockout mice could recapitulate disease features reminiscent of a biventricular form of human ARVC, (ii) myocardial tissue from human ARVC patients harboring DSP and PKP2 mutations displayed a reduction in CSN6 cell-cell junction localization, and (iii) genetic mouse models harboring DSP and PKP2 mutations showcased molecular and disease alterations disruptive of the DSP structural-CSN signaling axis (Liang et al. 2021). These data provide further validation to observational studies that mark the cardiac cell-cell junction as a resident site of protein degradation by the presence of ubiquitin conjugates, autophagosomes, and markers of ubiquitin-mediated selective autophagy at this location in cardiomyocyte health and disease states (Hilenski et al. 1992; Nepomnyashchikh et al. 2000; Lange et al. 2005; Balasubramanian et al. 2006; Hirschy et al. 2010).

## Arrhythmia mechanisms linking cardiac desmosome to channel function

The cardiac desmosome is classically thought to function as a cell-cell adhesive structure (Broussard et al. 2015); however, emerging evidence points to noncanonical roles for the cardiac desmosome in regulating electrical channels and function, independent of its classic structural roles. Crosstalk between desmosomes and neighboring gap junction protein, connexins, membrane-associated voltage-gated Na^+^ channels, and most recently calcium handling machinery has been postulated in driving cardiac arrhythmias independent of the structural remodeling response associated with desmosomal mutations/protein loss (Cerrone et al. 2012; Lyon et al. 2014; Mezzano et al. 2016; Cerrone et al. 2017; Kim et al. 2019).

### Cardiac desmosome crosstalk with connexins

**Fig. 2:**
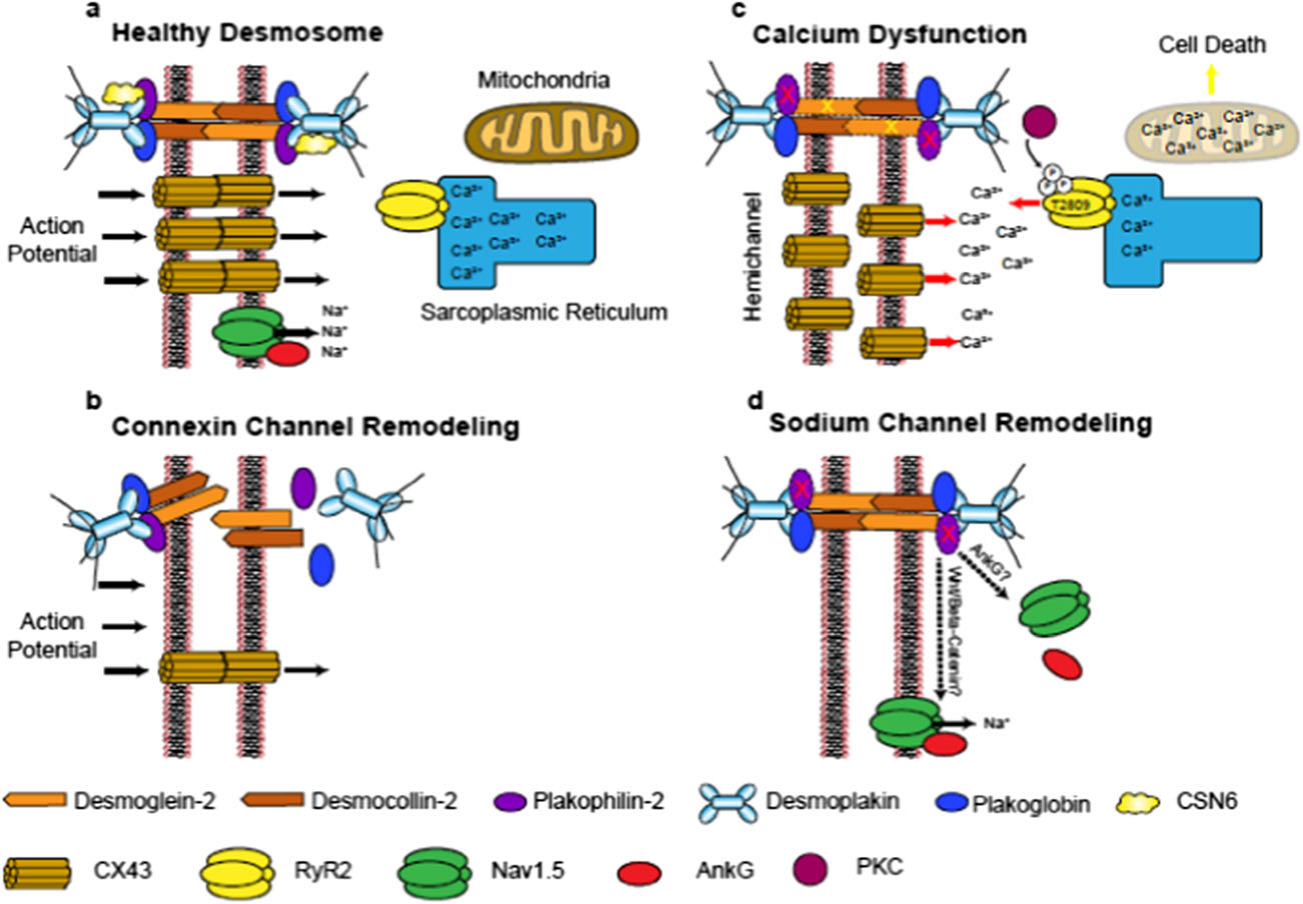
Arrhythmogenic pathways linked to desmosomal mutations and loss. (a) Healthy desmosomes enable proper connexin function for action potential propagation, calcium handling, and sodium channel activity. (b) Desmosomal loss triggers early connexin channel remodeling, which prevents proper electrical coupling between adjacent cardiomyocytes. (c) PKP2 loss causes calcium dysfunction through multiple mechanisms impacting connexin hemichannel permeability and PKC-driven increase in RYR2 activity (Ca^2+^ release from sarcoplasmic reticulum). (d) PKP2 missense mutations and loss can drive distinct mechanisms impacting sodium channel function, as well as localization

Gap junctions are primarily made up of connexins, which classically function as electrical channels connecting the cytoplasm of adjacent cardiomyocytes, and thus, allowing for intracellular transmission of ions (Ca^2+^, Na^2+^, etc.) and action potential propagation between cardiac muscle cells as a means to synchronize coordinated cardiac muscle contraction (Giovannone et al. 2012; Hoagland et al. 2019). Considerable attention has focused on the crosstalk between the cardiac desmosome and connexin43 (CX43), which is the major isoform identified in ventricular cardiomyocytes (Giovannone et al. 2012). Critical studies utilizing DSP-floxed neonatal mouse ventricular cardiomyocytes demonstrated early and dose-dependent loss of CX43 (and phosphorylation) following Cre-mediated dose-dependent DSP deletion, when compared to desmosomal structural proteins, such as PKP2 (Lyon et al. 2014). This reduction of CX43 triggered ventricular conduction abnormalities in an in vitro model system that would be independent of cardiomyocyte structural deficits (e.g., fibrofatty replacement) typically found within DSP-deficient hearts in vivo (Lyon et al. 2014), pointing to CX43 as an important early target of DSP loss and primary driver of electrical dysfunction. CX43 remodeling (aberrant expression and localization) is long been thought to be a molecular hallmark of hearts harboring the human desmosomal disease, ARVC (Fig. 2) (Asimaki et al. 2009; Fidler et al. 2009). However, electrical abnormalities (ventricular arrhythmias) associated with reduced CX43 cardiac cell junction localization could also be observed prior to fibro-fatty replacement in the myocardium in ARVC patients harboring DSP mutations (Gomes et al. 2012), extending this early crosstalk to the human myocardium. More recently, functional associations were identified between the cardiac desmosomal protein, DSP, and connexin45 (CX45), which is a predominant connexin isoform found in the cardiac sinus node (pacemaker) (Mezzano et al. 2014). Cardiac conduction system specific ablation of DSP resulted in loss of CX45 and resulting sinus node dysfunction as well as the identification of human DSP mutations in sinus bradycardia patients, further reinforced DSP loss as an early driver of connexin-mediated arrhythmias in the absence of cardiomyopathy (Mezzano et al. 2016). Direct crosstalk between desmocollin-2 and CX43 remodeling was also implicated as a novel human desmocollin-2a mutation associated with right ventricular arrhythmias was found to abrogate binding to the C-terminus of CX43 (Gehmlich et al. 2011). More recently, distinct connexin-mediated mechanisms were implicated in driving cardiac arrhythmias associated with PKP2 loss (Kim et al. 2019). PKP2 has been identified in CX43 plaques (Agullo-Pascual et al. 2013). However, recent work utilizing a mouse model with cardiac-specific and inducible deletion of PKP2 (PKP2cKO) in the adult heart, linked RV cardiomyocyte electrical abnormalities to increased CX43 hemichannel function leading to an increase in membrane permeability and intracellular calcium overload (Kim et al. 2019), rather than loss or abnormal localization of CX43 protein (Fig. 2). Studies focused on cross-breeding PKP2cKO mice with heterozygous cardiomyocyte CX43 knockout mice or pharmacological inhibition of hemichannel activity via TAT-Gap19 treatment could reduce membrane permeability and diastolic Ca^2+^ overload in PKP2cKO hearts, suggesting that increased hemichannel permeability following PKP2 loss could serve as an early arrhythmogenic substrate, and that this may be especially pertinent in adrenergic stress states (Kim et al. 2019).

### Cardiac desmosome crosstalk with calcium

It has long been known that Ca^2+^ signaling and especially Ca^2+^ overload is a trigger for cardiac arrhythmias (Landstrom et al. 2017); however, more recent studies have shed light into the role of desmosomal alterations in driving calcium mediated arrhythmogenic mechanisms (Kim et al. 2013; Cerrone et al. 2017; Kim et al. 2019). Studies in PKP2cKO mice-and PKP2 mutant-induced pluripotent stem cell-derived cardiomyocytes reinforced consequences on calcium dynamics as abnormal cardiomyocyte calcium handling and dysregulation of calcium handling gene expression could be observed (Kim et al. 2013; Cerrone et al. 2017; Kim et al. 2019). Studies in PKP2cKO cardiomyocytes at a young age further highlighted the right ventricular predominant nature of disruption in calcium homeostasis (Kim et al. 2019), which coincided with transcriptional downregulation of key components in calcium signaling pathways (RYR2, Ankyrin-B, Cav1.2) (Cerrone et al. 2017). Interestingly, calcium dysregulation in hearts of PKP2cKO mice occurred independent of CX43 remodeling and voltage-gated Na^+^ channel subunit Nav1.5 remodeling but could be blunted with protein kinase C inhibition (Kim et al. 2019). Through this study, the authors hypothesized a multi-pronged mechanism, whereby PKP2 loss impacted desmosomal integrity and thus, disrupted cell-cell junction resident proteins such as neighboring connexins (increased hemichannel activity) as well as protein kinase C, which was now cytosolic bound and free to phosphorylate off-target sites on RYR2 (T2809) and drive calcium channel dysregulation and overload (Fig. 2) (Kim et al. 2019). Interestingly, recent studies have drawn a connection between the desmosomal protein, DSG2, and mitochondrial-mediated calcium overload in ARVC in exercise settings via the calpain-1 pathway and shown that its inhibition can circumvent cardiomyocyte death by interfering with cleavage of apoptosis inducing factor (Chelko et al. 2021). These latter studies reveal the possibility that mitochondrial calcium overload induced cell death may generate arrhythmogenic substrates and further contribute to arrhythmias.

### Cardiac desmosome crosstalk with sodium channels

Nanoscale imaging microscopy studies have demonstrated the localization and functional relevance of the sodium channel, Nav1.5, at the cardiac cell-cell junction (Leo-Macias et al. 2016), suggesting the potential for crosstalk between the cardiac desmosome and sodium channel activity. Although *SCN5A* mutations (gene coding for Nav1.5) are directly linked to the inherited cardiac arrhythmia, Brugada syndrome (Wilde and Amin 2018), a retrospective analysis of Brugada patients identified that a subgroup of these patients harbored missense mutations in PKP2 (Cerrone et al. 2014). Further cardiac analyses of PKP2-deficient models revealed an impact on sodium channel activity and cell adhesion defects that may be driven by microtubule plus ends (Cerrone et al. 2014), providing further validation to the desmosome-sodium channel relationship. Relevance to humans was shown as subsequent studies identified *SCN5A* mutations in a subpopulation of patients harboring the desmosomal disease, ARVC (Te Riele et al. 2017). In addition, reduced immunoreactive cardiac Nav1.5 levels were observed in a majority of ARVC patients (Fig. 2) (Noorman et al. 2013). Studies focused on the adaptor protein, ankyrin G (AnkG), also revealed its functional relevance at the cardiac cell-cell junction and sodium channel activity (Makara et al. 2014). Canonical functions of ankyrins include the ability to localize ion channel and transporters to the membrane (Cunha and Mohler 2009). Early studies by Delmar and colleagues showcased a complex interaction between PKP2, AnkG, and CX43 in regulating cardiomyocyte Nav1.5 remodeling and sodium channel activity using in vitro model systems (Sato et al. 2011). Reduced Nav1.5 expression and sodium channel activity has been observed in conditional CX43 heterozygous deficient hearts that harbored arrhythmia vulnerability (Jansen et al. 2012). More recent work has highlighted a role for AnkG to target both the Nav1.5 and the sarcolemmal ATP-sensitive K^+^ (K_ATP_) channel to the cardiac intercalated disc (Yang et al. 2020). Specificity of these interactions was interrogated through interfering with AnkG binding sites (competitive peptides and patient mutation) on these genes, which appears to be a mechanism for functional coupling between these channels (Yang et al. 2020). Recent studies by Mohler and colleagues identified rare variants in ankyrin B in patients harboring desmosomal disease, ARVC, as well as a molecular link to the cardiac cell-cell junction via beta-catenin as targeted inhibition of the Wnt/beta-catenin pathways (SB-216763) could prevent and partially rescue ARVC phenotypes in cardiac-specific ankyrin B (*Ank2*)-deficient mice, revealing arrhythmogenic pathways in mice distinct from classic desmosomal structural alterations and remodeling (Roberts et al. 2019). Independent studies utilizing human PKP2 mutant iPSC-derived cardiomyocytes further highlighted a role for Wnt/beta-catenin pathway in regulating sodium channel activity (Khudiakov et al. 2020). Downregulation of Wnt/beta-Catenin signaling activity, and a corresponding reduction in sodium current density was observed in PKP2 mutant iPSC-derived cardiomyocytes, despite normal levels and localization of Nav1.5 (Khudiakov et al. 2020). Restoration of Wnt/beta-Catenin signaling (SB-216763) can alleviate sodium current defects, implying that this pathway may be an important modulator of Nav1.5 activity (Fig. 2) (Khudiakov et al. 2020). These studies may provide further validation to studies performed in PKP2-deficient cardiomyocytes, PKP2 heterozygous KO mice, and DSG2 mutant transgenic mice that displayed reduced sodium current function in the absence of changes to Nav1.5 localization or levels (Sato et al. 2009;Cerrone et al. 2012; Rizzo et al. 2012). These studies altogether showcase that Nav1.5 channel function and crosstalk to molecular complexes at the cardiac cell junction may be an important contributor to electrical dysfunction following cell-cell (desmosomal) junction disruption, as well as may be a target for intervention.

## Cardiac desmosomal dysfunction and inflammatory pathways

**Fig. 3:**
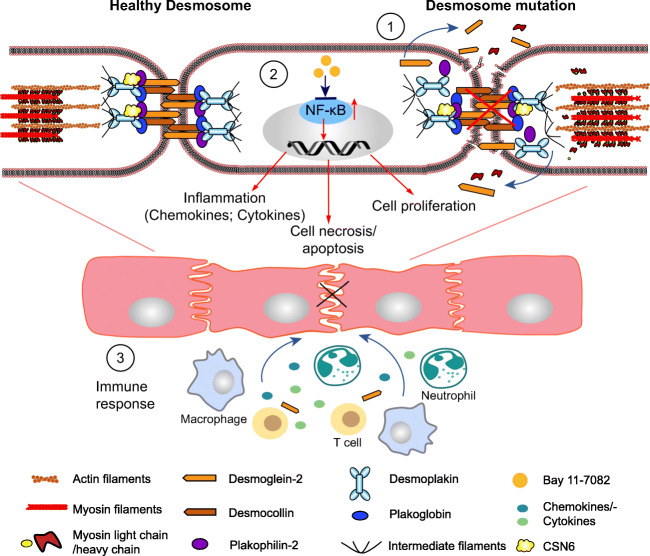
Schemata of inflammatory response associated with ARVC. (1) Within healthy desmosomes, two adjacent cardiac cells are tightly connected. In the setting of desmosomal mutations, truncated/fragmented desmosomal proteins start to fall apart from cell membranes, which leads to the cell death and release of specific antigens (e.g., unmasked DSG2). (2) In the setting of desmosomal mutations/loss, inflammatory pathways, such as NF-κB signaling is activated, resulting in upregulation of target gene expression and chemokine/cytokine secretion, activation of cell apoptosis/necrosis, and cell proliferation. Small molecular Bay 11-7082 could inhibit the activation of NF-κB and reverse the disease progression. (3) The immune activation includes release of chemokines/cytokines, recruitment of immune cell types (e.g., neutrophils, T-cell, and macrophage) targeted to cardiomyocytes. Inflammatory infiltration further triggers cardiac cell death and fibrotic scar formation

There has been a longstanding connection between the cardiac desmosome and inflammatory pathways at the clinical level, as early stages of arrhythmogenic right and left ventricular cardiomyopathies, 40-50% of which are associated with desmosomal gene mutations, are virtually clinically indistinguishable from the cardiac inflammatory syndrome, myocarditis (Basso et al. 1996; Pieroni et al. 2009). A deeper dive into the immune response reveals connections, in some cases, between ARVC and loss of desmosomal cell-cell junction integrity and the appearance of unmasked cardiac cell and junctional related epitopes (due to desmosomal gene mutations), which are recognized as self-antigens (auto-antigens) by immune cells and mistakenly targeted to generate autoimmune antibodies (Chatterjee et al. 2018; Lin et al. 2021). Downstream signaling mediators of inflammation, such as NF-κB, are also integral to the ARVC inflammatory response as immunomodulatory therapies targeted at inhibiting NF-κB signaling show functional improvements in a DSG2 mutant-based mouse model harboring ARVC disease features (Chelko et al. 2019) (Fig. 3).

### Myocarditis and autoantibody presence as a consequence of an ARVC heart

Studies first identified a connection between early ARVC pathogenesis and myocarditis, as the disease etiology of myocarditis encompassed selective right ventricular defects, which associated to right ventricular electrophysiological defects (inverted T wave in right precordial leads) resulting in ventricular arrhythmias (ventricular tachycardia) that could be indistinguishable from proven ARVC patients (Pieroni et al. 2009). The presence of lymphocyte infiltrates have also been observed in the fibrofatty patches in human ARVC hearts (Basso et al. 1996; Campuzano et al. 2012). Studies in a transgenic mouse model of ARVC expressing a *DSG2*-N266S mutation suggested that cardiomyocyte death (necrosis) may be an initiating factor to this inflammatory response (Pilichou et al. 2009); however, the triggers and cause-effect relationship remains unexplained and an intense area of investigation. Human genetic studies have provided added insight and identified human DSP variants to have a particular affinity to intermittent myocardial inflammatory episodes similar to myocarditis but encompassing a distinct cardiomyopathy associated with left ventricular dominant fibrosis and arrhythmias (Smith et al. 2020). Interestingly, ARVC patients with PKP2 mutations in this study, for which right ventricular dysfunction was a stronger predictor, were shown not to associate with these inflammatory episodes (Smith et al. 2020), revealing a potential desmosomal gene-specific connection that favored the appearance of episodic cardiac inflammatory events in humans. A recent study further associated women being especially vulnerable to the initiating presence of clinical myocarditis in ARVC that harbored left ventricular involvement and prevalence of pathogenic DSP variants (Scheel et al. 2021).

Despite evidence highlighting the occurrence and association of clinical myocarditis in ARVC, there is limited evidence to suggest the dominant or direct involvement of enteroviruses as a cause (Grumbach et al. 1998; Calabrese et al. 2000). Instead, a growing body of evidence support a pathogenic autoimmune response associated with the presence of autoantibodies that may underlie virus-negative myocarditis in ARVC (Chatterjee et al. 2018;Caforio et al. 2020). ARVC probands were found to express autoimmune biomarkers, which include organ specific and disease specific serum anti-heart autoantibodies (AHAs) and anti-intercalated disc autoantibodies (AIDAs) as well as desmosomal targeted anti-DSG2 autoantibodies at a higher level when compared to healthy controls (Chatterjee et al. 2018; Caforio et al. 2020). To further showcase the specificity of these responses to ARVC, it was shown that these autoantibodies were absent or not increased in another heart muscle disease, hypertrophic cardiomyopathy, where no myocardial inflammation was observed at the histopathological level (Chatterjee et al. 2018; Caforio et al. 2020). Interestingly, no clear associations were observed between desmosomal mutation type and the level of autoantibodies (Chatterjee et al. 2018; Caforio et al. 2020). However, a correlation to disease severity was observed as ARVC patients with a high level of anti-AHA and anti-AIDA antibodies displayed worsened electrical dysfunction and lower ejection fraction (Caforio et al. 2020). In addition, high levels of anti-DSG2 autoantibodies correlated with a higher burden of premature ventricular contractions in ARVC patients (Chatterjee et al. 2018), altogether highlighting their potential as inflammatory biomarkers in ARVC.

### Inflammatory pathways impacted in ARVC mouse models and patients

Based on the presence of anti-DSG2 autoantibodies in ARVC patients (Chatterjee et al. 2018), there has been significant effort in understanding specific immune cell populations and chemokines that drive the cardiac inflammatory response in ARVC using DSG2 models that harbor ARVC disease features and either express a mutant truncated form of DSG2 or cardiomyocyte restricted DSG2 loss (Lubos et al. 2020). These studies altogether revealed a focal neutrophil dominant inflammatory response confined to ARVC-related fibrofatty patches that was initiated by cardiomyocyte necrosis (Lubos et al. 2020). The mRNA levels of chemokines ccl2 and ccl3 as well as their relative receptors were found to be significantly upregulated in DSG2 mutant mice (Lubos et al. 2020). Interestingly, ccl3 was found to be upregulated even when macroscopically visible lesions were not observed (Lubos et al. 2020). Aside from the activation of many chemokines/cytokines, they also showed an involvement of macrophages and T cells in the formation of the fibrotic and calcified scar during both acute and chronic ARVC disease states (Lubos et al. 2020).

Connections between the desmosomal gene, PKP2, and inflammatory pathways were also recently highlighted through the characterization of the inflammatory transcriptome in cardiac-specific inducible PKP2 knockout mice (PKP2-cKO) crossed to the Ribotag mouse as a means to identify ribosome-resident transcriptionally changes in inflammation in cardiomyocytes (Perez-Hernandez et al. 2020). RNA sequencing analyses revealed that platelet activation, chemokine signaling, viral response, and immune/inflammatory pathways were highly upregulated in PKP2-cKO mice (Perez-Hernandez et al. 2020). Specific pathways included C-type lectin receptor signaling, NFκB, REL, and EGR1, transcription factors related with Th17 differentiation and immune response (Perez-Hernandez et al. 2020); however, further work will be needed to better understand to how they directly intersect with PKP2. These studies further identified immune cell infiltration in subepicardial region of PKP2-cKO hearts, at a stage where no apparent histological changes could be observed (Perez-Hernandez et al. 2020), highlighting subpopulations of cells that may be particularly prone to inflammation.

NF-κB signaling pathway is a central mediator of inflammation and implicated in the pathogenesis of various inflammatory diseases, such as rheumatoid arthritis, multiple sclerosis, atherosclerosis, and cancer (Liu et al. 2017; Taniguchi and Karin 2018). Recent studies have demonstrated nuclear factor-κB signaling as a critical modulator of the immune response underlying ARVC disease progression (Chelko et al. 2019). In studies performed in neonatal rat cardiomyocytes expressing a deletion mutant of JUP, hearts from a DSG2 mutant model harboring a truncated DSG2 and PKP2 mutant human-induced pluripotent stem cell-derived cardiomyocytes demonstrated high levels of secretion of various inflammatory mediators that could be significantly blunted by NF-κB inhibition via Bay 11-7082 (Chelko et al. 2019). Interestingly, ARVC patients also show higher plasma levels of inflammatory cytokines (Campian et al. 2010)**.** In addition, ARVC disease phenotypes in the DSG2 mutant mouse model could also be dampened by NF-κB inhibition via Bay 11-7082 (Chelko et al. 2019), further demonstrating inflammatory modulators as potential therapeutic targets for ARVC.

## Conclusions

Although the cardiac desmosome is thought to have canonical functions as a structural element in the cardiomyocyte, recent studies have highlighted new resident regulatory functions that relate to its protein turnover as well as cross talk with electrical channels as well as inflammatory cell types and pathways. At least two pathways have been linked to cardiac desmosomal protein homeostasis, one which is resident (CSN6) to the desmosome and one found in the cytosol (calpain); however, future studies should focus on how these pathways work cooperatively to degrade desmosomal proteins in the context of human ARVC-associated desmosomal mutations. Desmosomes are an essential hub for electrical homeostasis in the cardiomyocyte. Recent work highlights that diverse arrhythmogenic pathways may relate to specific desmosmomal proteins with PKP2 mutations/loss (CX43 hemichannel function, calcium handling, sodium channels) while other desmosomal proteins, such as DSP may impact classic CX43 channel functions, which may drive electrical dysfunction in the absence of structural disease in ARVC. Further work is required to dissect whether all desmosomal proteins equally impact these distinct channel functions or whether there are adapter proteins associated with the desmosome that may uniquely mediate desmosome-associated arrhythmias. Additional findings suggest that inflammatory signaling pathways may play an important role in the pathogenesis of ARVC associated with desmosomal mutations. Early infiltration of immune cells and autoantibodies provide new evidence for better clinical diagnosis of ARVC; however, there remains limited direct evidence of the relationship (crosstalk) between desmosomal loss/deficiency and recruitment of immune cells in ARVC progression, which should be a focus for future studies.
